# Australian Emergency Department Doctors and Nurses' Perspectives on the Duration of Persistent Tachycardia in Children

**DOI:** 10.1111/1742-6723.70118

**Published:** 2025-08-07

**Authors:** Anastasia Mutic, Eunicia Tan, Michael Fahey, Emily Callander, Libby Haskell, Shane George, Meredith Borland, Naomi Loftus, Jessica Kasza, Jeremy Furyk, Natalie Phillips, Stuart R. Dalziel, Simon Craig, the PREDICT Network

**Affiliations:** ^1^ Department of Paediatrics Monash University Clayton Victoria Australia; ^2^ Department of Surgery, Faculty of Medical and Health Sciences The University of Auckland Auckland New Zealand; ^3^ Emergency Department Middlemore Hospital Auckland New Zealand; ^4^ Department of Paediatric Neurology Monash Health Clayton Victoria Australia; ^5^ University of Technology Sydney, School of Public Health Sydney Australia; ^6^ Children's Emergency Department Starship Children's Hospital Auckland New Zealand; ^7^ Department of Paediatrics, Child and Youth Health, Faculty of Medical and Health Sciences The University of Auckland Auckland New Zealand; ^8^ Department of Emergency Medicine Gold Coast University Hospital Southport Queensland Australia; ^9^ School of Medicine and Dentistry, Griffith University Southport Queensland Australia; ^10^ Child Health Research Centre The University of Queensland Brisbane Queensland Australia; ^11^ Emergency Department, Perth Children's Hospital Nedlands Western Australia Australia; ^12^ Divisions of Emergency Medicine and Paediatrics, School of Medicine University of Western Australia Crawley Western Australia Australia; ^13^ Emergency Department Monash Medical Centre Clayton Victoria Australia; ^14^ School of Public Health and Preventive Medicine Monash University Melbourne Victoria Australia; ^15^ University Hospital Geelong, Barwon Health Geelong Victoria Australia; ^16^ School of Medicine, Deakin University Geelong Victoria Australia; ^17^ Queensland Children's Hospital Brisbane Queensland Australia

## Abstract

**Objective:**

To explore time thresholds for ‘persistent tachycardia’ in children among Australian emergency department clinicians.

**Methods:**

Online cross‐sectional survey of emergency department clinicians. Respondents were asked to indicate the duration in hours they considered that a tachycardia in a child would be classified as ‘persistent’.

**Results:**

Among 499 respondents, 304 (60.9%) identified tachycardia as ‘persistent’ by 2 h, and 471 (94.3%) by 4 h; the most common response was 2 h (147 (51.2%) doctors; 78 (36.8%) nurses).

**Conclusions:**

Time based thresholds for ‘persistent tachycardia’ differ. This has implications for its use in rapid‐response systems and early recognition of serious illness.

## 1 Introduction

The presence of ‘persistent tachycardia’ in children is commonly suggested as an early warning sign for potential serious illnesses such as sepsis. Persistent tachycardia has been recently identified as the most common feature in paediatric cases involving critical illness and death, occurring in 60% of cases [1]. Despite common usage, there is no clear definition.

Current guidelines, including those published by the National Institute for Health and Care Excellence [2], Royal Children's Hospital Melbourne [3], and New South Wales (NSW) Clinical Excellence Commission [4], emphasise *persistent* tachycardia as a key indicator of serious illness and a tool for identifying sepsis in febrile infants and children. However, the absence of a defined duration for tachycardia limits its practical applicability, leaving it unclear how long an elevated heart rate must persist to warrant clinician concern or need for intervention. This ambiguity can impact rapid response system activation and lead to variability in diagnosis and management practices.

## 2 Objective

To assess emergency doctors' and nurses' threshold for the duration of elevated heart rate they consider as ‘persistent tachycardia’ in a paediatric population.

## 3 Methods

A cross‐sectional survey of doctors and nurses was conducted across 22 Australian emergency departments (EDs) within the PREDICT (Paediatric Research in Emergency Departments International CollaboraTive) network. Eligible participants were full‐ or part‐time staff working at least one shift per week (on average) in an ED treating children < 2 years of age. Exclusion criteria included junior medical staff (postgraduate year two or less), temporary nursing agency staff, or medical locums.

The survey obtained information on: participant information and consent, demographics, questions relating to fever management and antipyretic use, and threshold of ‘persistent tachycardia’ duration (Box 1).

BOX 1 **Question related to the definition of persistent tachycardia.**

*Some clinicians consider* “*persistent tachycardia*” *is a risk factor for more serious illness*.

***In your opinion, how many hours should tachycardia last before it is considered to be persistent tachycardia?***

– 1 hour
– 2 hours
– 3 hours
– 4 hours
– 5 hours
– 6 hours
– 7 hours
– 8 hours
– 9 hours
– 10 hours
– 11 hours
– 12 hours

The survey was deemed exempt from formal ethics committee review by Monash Health Human Research Ethics Committee (RES23‐0000‐780Q). The online survey was accessed and completed anonymously via a link distributed by nominated PREDICT members and required 10–15 min to complete. Data were collected and managed using a REDCap electronic data capture tool hosted and managed by Helix (Monash University).

Data were analysed using SPSS Statistics 29. Counts and percentages were used to describe demographic characteristics. Pearson Chi‐squared tests were used to compare differences in demographic characteristics and thresholds for persistent tachycardia between doctors and nurses.

## 4 Results

Between March and May 2024, the survey was sent to 818 ED clinicians, with 499 responses received; response rate of 61.0% (doctors 287/344 [83.4%]; nurses 212/305 [69.5%]). Nearly half of respondents were employed at a major referral centre (230/473 [48.6%]) (Table 1). Compared to their nursing colleagues, more doctors had over 5 years of professional experience (93.4% vs. 81.6%, *p* < 0.001) and a higher percentage held a senior clinical role (63.1% vs. 44.8%, *p* < 0.001).

**TABLE 1:** Participant characteristics.

	All participants		Doctors		Nurses	
	*N*	*n* (%)	*N*	*n* (%)	*N*	*n* (%)
Type of ED (ACEM designation)	473		276		197	
Major referral		230 (48.6)		144 (52.2)		86 (43.7)
Urban district		98 (20.7)		45 (16.3)		53 (26.9)
Regional referral/other		145 (30.7)		87 (31.5)		58 (29.4)
Years of experience in profession	499		287		212	
0–4		58 (11.6)		19 (6.6)		39 (18.4)
5–9		120 (24.0)		65 (22.6)		55 (25.9)
10–14		141 (28.3)		84 (29.3)		57 (26.9)
≥ 15		180 (36.1)		119 (41.5)		61 (28.8)
Clinical role—senior	499	276 (55.3)	287	181 (63.1)	212	95 (44.8)
Paediatric‐specific qualifications	498	146 (29.3)	286	92 (32.2)	212	54 (25.5)

*Note:* Missing data for the following: type of ED (*n* = 26), paediatric‐specific qualifications (*n* = 1). Senior doctor role includes consultant, fellow; senior nurse role includes advanced practice nurse (nurse practitioner and nurse specialist), nurse educator, charge nurse. Paediatric‐specific qualifications include Fellowship in paediatrics, subspecialty ACEM training in paediatric emergency medicine, Nursing Masters.

Abbreviations: ACEM, Australasian College for Emergency Medicine; ED, emergency department.

The threshold of persistent tachycardia duration varied between doctors and nurses, with more doctors preferring shorter durations than nurses. Two hours was the most common response, selected by 225 (45.1%) of respondents, and was more frequently selected by doctors (doctors 147/287 [51.2%], nurses 78/212 [36.8%], difference 14.4% [95% CI 5.6%–23.0%], *p* = 0.001). Few respondents selected durations of 5 to 12 h (28 5.6%) or a duration of 1 h (79, 15.8%).

**FIGURE 1:**
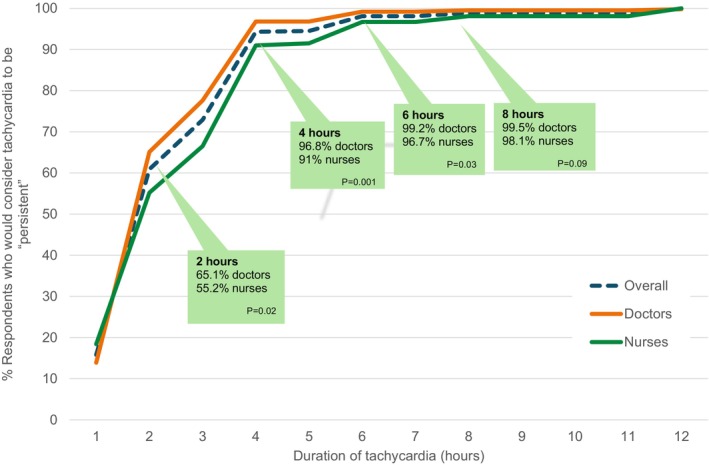
Cumulative thresholds for persistent tachycardia. *p* values refer to the Chi‐square test that there is no difference in the cumulative proportions of doctors and nurses who selected this value or lower as the duration to define persistent tachycardia.

Cumulative thresholds are shown in Figure 1. By 4 h, 471 (94.4%) of overall respondents determined that a tachycardia would be considered persistent. Cumulative thresholds are shown in Figure 1.

## 5 Discussion

Thresholds for ‘persistent tachycardia’ in children among Australian ED clinicians differ but fall mostly within 2–4 h. Clinical context remains very important: treatment aimed solely at normalising an isolated elevated heart rate may not improve patient outcomes or reduce adverse events [5].

The lack of a clear definition for persistent tachycardia poses potential clinical and medicolegal risks. Any agreed definition should be based on important clinical outcomes rather than consensus surveys. Future research should investigate the relationship between the clinician‐defined thresholds of persistent tachycardia with clinically significant outcomes such as sepsis, septic shock, intensive care unit (ICU) admission and death, and the possible incorporation of persistent tachycardia in rapid‐response systems and early warning tools. Exploring whether tachycardia following antipyretic treatment requires a different diagnostic approach would also be valuable.

Limitations of this study include its focus on ED clinicians, excluding inpatient clinicians whose perspectives may differ. Strengths include response survey rate and wide inclusion of clinicians from different ED settings reflective of where children present.

## 6 Conclusions

Threshold for ‘persistent tachycardia’ in children among Australian ED clinicians is generally within 2–4 h. Further research is required to determine a threshold of persistent tachycardia relevant to clinical outcomes, activation of rapid‐response systems and early recognition of serious illness.

## Conflicts of Interest

Simon Craig and Shane George are section editors of *Emergency Medicine Australasia*.

## Data Availability

The data that support the findings of this study are available from the corresponding author upon reasonable request.

## References

[1] P. Aldridge , A. J. Baldock , J. Baird , A. Elson , and S. McGregor , “Recurrent Themes From Paediatric Mortality and Morbidity: A Network Perspective 2021‐2023,” Archives of Disease in Childhood 109, no. 4 (2024): 354–355. 38233097 10.1136/archdischild-2023-326633

[2] National Institute for Health and Care Excellence. National Institute for Health and Care Excellence: Guidelines. Fever in Under 5s: Assessment and Initial Management (National Institute for Health and Care Excellence (NICE) Copyright NICE 2021, 2021). 31891472

[3] Royal Children's Hospital , “Febrile Child 2022,” https://www.rch.org.au/clinicalguide/guideline_index/febrile_child/.

[4] Clinical Excellence Commission , “Paediatric Watch—Lessons From the Frontline: Sepsis in a Heartbeat,” 3/17 ed2022.

[5] D. Roland and E. Snelson , “So Why Didn't You Think This Baby Was Ill?' Decision‐Making in Acute Paediatrics,” Archives of Disease in Childhood. Education and Practice Edition 104, no. 1 (2019): 43–48. 29496733 10.1136/archdischild-2017-313199

